# Recent Advances in the Preparation Methods of Magnesium-Based Hydrogen Storage Materials

**DOI:** 10.3390/molecules29112451

**Published:** 2024-05-23

**Authors:** Yaohui Xu, Yang Zhou, Yuting Li, Yechen Hao, Pingkeng Wu, Zhao Ding

**Affiliations:** 1 Laboratory for Functional Materials, School of New Energy Materials and Chemistry, Leshan Normal University, Leshan 614000, China; 2 State Key Laboratory of New Textile Materials and Advanced Processing Technology, School of Textile Science and Engineering, Wuhan Textile University, Wuhan 430200, China; 3 Leshan West Silicon Materials Photovoltaic New Energy Industry Technology Research Institute, Leshan 614000, China; 4 College of Materials Science and Engineering, National Engineering Research Center for Magnesium Alloys, National Innovation Center for Industry-Education Integration of Energy Storage Technology, Chongqing University, Chongqing 400044, China; 5 Department of Computer Science, Illinois Institute of Technology, Chicago, IL 60616, USA; 6 Department of Chemical Engineering, Illinois Institute of Technology, Chicago, IL 60616, USA

**Keywords:** magnesium-based materials, hydrogen storage, preparation methods, ball milling, nanostructures, catalysts, kinetics, thermodynamics

## Abstract

Magnesium-based hydrogen storage materials have garnered significant attention due to their high hydrogen storage capacity, abundance, and low cost. However, the slow kinetics and high desorption temperature of magnesium hydride hinder its practical application. Various preparation methods have been developed to improve the hydrogen storage properties of magnesium-based materials. This review comprehensively summarizes the recent advances in the preparation methods of magnesium-based hydrogen storage materials, including mechanical ball milling, methanol-wrapped chemical vapor deposition, plasma-assisted ball milling, organic ligand-assisted synthesis, and other emerging methods. The principles, processes, key parameters, and modification strategies of each method are discussed in detail, along with representative research cases. Furthermore, the advantages and disadvantages of different preparation methods are compared and evaluated, and their influence on hydrogen storage properties is analyzed. The practical application potential of these methods is also assessed, considering factors such as hydrogen storage performance, scalability, and cost-effectiveness. Finally, the existing challenges and future research directions in this field are outlined, emphasizing the need for further development of high-performance and cost-effective magnesium-based hydrogen storage materials for clean energy applications. This review provides valuable insights and references for researchers working on the development of advanced magnesium-based hydrogen storage technologies.

## 1. Introduction

Hydrogen, as a clean and renewable energy carrier, has attracted increasing attention due to its potential to mitigate environmental issues associated with fossil fuels and address growing energy demands [1]. However, the safe and efficient storage of hydrogen remains a major challenge for its widespread application. Among various hydrogen storage methods, solid-state hydrogen storage materials have emerged as promising candidates owing to their high volumetric and gravimetric hydrogen storage densities, safety, and reversibility [2].

Magnesium-based hydrogen storage materials have been extensively investigated due to their high theoretical hydrogen storage capacity (7.6 wt.% for MgH_2_), abundance, and low cost, positioning them as promising candidates for realizing a sustainable and clean energy future [3,4]. The successful development of these materials could significantly promote the implementation of sustainable and clean energy, reduce greenhouse gas emissions, and decrease dependence on fossil fuels.

Despite the significant theoretical advantages of magnesium-based hydrogen storage materials, their practical application still faces several critical technical challenges. Firstly, the high thermodynamic stability of magnesium hydride requires temperatures exceeding 300 °C for effective hydrogen release, limiting its use in low-temperature applications [5,6]. Additionally, the kinetics of hydrogen absorption and release are slow, primarily due to the strong ionic bonding between magnesium and hydrogen, limited hydrogen diffusion through the hydride layer, and high activation energy required for hydrogen dissociation and recombination. These factors collectively result in the suboptimal performance of magnesium-based hydrogen storage materials in rapid charging and discharging, thereby restricting their widespread use in high-demand energy conversion systems [7].

To address these challenges, researchers have developed various innovative material preparation techniques to enhance the hydrogen storage performance of magnesium-based materials. Traditional methods such as mechanical ball milling can effectively reduce particle size and increase the reaction surface area but may introduce impurities, affecting the material’s performance and stability. Therefore, researchers have explored advanced techniques, including chemical vapor deposition, plasma-assisted ball milling, and organic ligand-assisted synthesis. These methods not only control the morphology and structure of the materials but also introduce defect sites or catalysts to reduce the energy barriers for hydrogen dissociation and recombination, thereby accelerating the rates of hydrogen absorption and release [8,9]. Recent research findings indicate that these advanced preparation techniques significantly improve the hydrogen absorption and release efficiency of magnesium-based materials, demonstrating their immense potential in hydrogen energy applications.

This review focuses on the latest preparation methods for magnesium-based hydrogen storage materials, including mechanical ball milling, methanol-wrapped chemical vapor deposition, plasma-assisted ball milling, and organic ligand-assisted synthesis. These methods have demonstrated considerable potential in enhancing the hydrogen storage performance of magnesium-based materials and are at the forefront of current research. We will discuss in detail the principles, processes, key parameters, and modification strategies of each method; evaluate and compare the advantages and disadvantages of different preparation methods; and analyze their impact on hydrogen storage properties. Additionally, we will assess the practical application potential of these methods, considering factors such as hydrogen storage performance, scalability, and cost-effectiveness. Finally, existing challenges and future research directions in this field will be outlined.

## 2. Basic Characteristics

### 2.1. Thermodynamic Properties

The thermodynamic properties of magnesium-based hydrogen storage materials, such as the enthalpy and entropy of hydrogen absorption and desorption, determine the equilibrium pressure and temperature conditions for hydrogen storage. The enthalpy of the formation of magnesium hydride (MgH_2_) is approximately −75 kJ/mol H_2_, which results in a high desorption temperature (>300 °C) at atmospheric pressure [10]. This high thermodynamic stability is attributed to the strong ionic bonding between magnesium and hydrogen atoms. Reducing the thermodynamic stability of MgH_2_ is essential for lowering the desorption temperature and improving the hydrogen storage performance.

The entropy change during the hydrogen absorption and desorption processes also plays a crucial role in determining the equilibrium conditions. The entropy change for the formation of MgH_2_ is approximately −130 J/(mol·K), which contributes to the high equilibrium pressure required for hydrogen desorption at a given temperature [11]. Strategies to modify the thermodynamic properties of magnesium-based materials, such as alloying with other elements or incorporating catalytic additives, can help to reduce the desorption temperature and improve the overall hydrogen storage performance.

### 2.2. Kinetic Properties

The kinetic properties of magnesium-based hydrogen storage materials, including the hydrogen absorption and desorption rates, are crucial for practical applications. The slow kinetics of magnesium hydride are attributed to the limited diffusion of hydrogen atoms through the hydride layer and the high activation energy for hydrogen dissociation and recombination [12,13]. The formation of a stable hydride layer on the surface of magnesium particles during the absorption process can hinder further hydrogen diffusion, leading to slow absorption kinetics. Similarly, the desorption process is limited by the slow recombination of hydrogen atoms and the diffusion of hydrogen through the hydride layer.

To improve the kinetic properties of magnesium-based materials, various strategies have been employed, such as reducing particle size, increasing surface area, creating defects, and introducing catalytic additives [14]. These strategies aim to enhance the hydrogen diffusion pathways, increase the number of active sites for hydrogen dissociation and recombination, and lower the activation energy barriers.

### 2.3. Cycling Stability

The cycling stability of magnesium-based hydrogen storage materials refers to their ability to maintain their hydrogen storage capacity and kinetic properties over multiple absorption–desorption cycles. The degradation of hydrogen storage performance during cycling is often caused by the sintering and aggregation of magnesium particles, the formation of stable oxide layers, and the contamination of impurities [15,16]. Sintering occurs due to the high temperatures and pressures involved in the hydrogen absorption and desorption processes, leading to a reduction in surface area and a decrease in the number of active sites. The formation of stable oxide layers on the surface of magnesium particles can also hinder hydrogen diffusion and limit the reversibility of the hydrogen storage process.

Improving the cycling stability of magnesium-based materials is essential for their long-term use in hydrogen storage applications. Strategies to enhance cycling stability include the incorporation of nanostructured support materials, such as carbon nanotubes or graphene, which can prevent particle sintering and aggregation [17]. Surface modification techniques, such as the application of protective coatings or the introduction of oxygen barrier layers, can also help to mitigate the formation of stable oxide layers and improve cycling stability [18].

### 2.4. Other Important Properties

Table 1 summarizes other important properties of magnesium-based hydrogen storage materials including their structural and morphological characteristics, such as particle size, surface area, and crystallinity, which can significantly influence their hydrogen storage performance [19]. Smaller particle sizes and higher surface areas generally lead to improved kinetic properties, as they provide shorter diffusion paths and more active sites for hydrogen absorption and desorption. The crystallinity of magnesium-based materials can also affect their hydrogen storage properties, with amorphous or nanocrystalline structures often exhibiting enhanced kinetics compared to their crystalline counterparts [20].

The thermal conductivity of magnesium-based materials is another important factor to consider, as it can influence the heat management in hydrogen storage systems. Magnesium hydride has a relatively low thermal conductivity, which can lead to heat accumulation during the absorption process and hinder the heat removal during the desorption process [24]. Improving the thermal conductivity of magnesium-based materials, for example, by incorporating high-conductivity additives or optimizing the system design, can help to enhance the overall hydrogen storage performance.

The mechanical properties of magnesium-based materials, such as their hardness, strength, and durability, are also crucial for their practical application in hydrogen storage systems. The repeated absorption and desorption cycles can induce mechanical stresses and strains in the materials, leading to degradation and failure over time [25]. Enhancing the mechanical properties of magnesium-based materials, through techniques such as compositing with reinforcing agents or optimizing the microstructure, can improve their long-term stability and reliability in hydrogen storage applications. 

## 3. Preparation Methods

### 3.1. Mechanical Ball Milling

#### 3.1.1. Principles and Processes

Mechanical ball milling is a widely used technique for the preparation of magnesium-based hydrogen storage materials. The process involves the repeated welding, fracturing, and rewelding of powder particles in a high-energy ball mill, which leads to the reduction in particle size, the creation of fresh surfaces, and the introduction of defects and strains [26]. The ball-milling process can also facilitate the formation of metastable phases and the dispersion of catalytic additives, which can enhance the hydrogen storage properties of magnesium-based materials.

**Figure 1:**
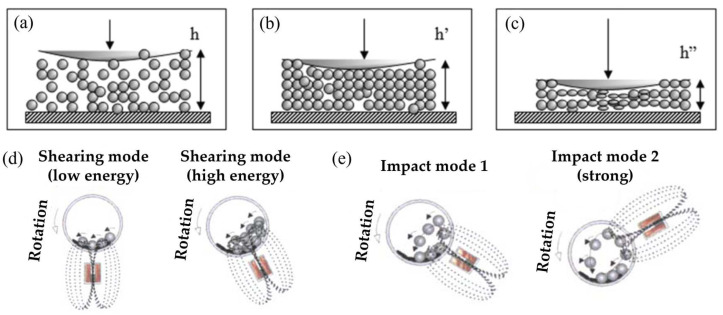
(**a**–**c**) Illustration of the deformation of powder agglomerate during the impact process [28]; strong external magnets induce (**d**) high-energy shearing (HES), (**e**) intense impact (IMP2) milling modes [30].

During the ball-milling process, the powder particles are subjected to high-energy collisions with the milling balls and the walls of the milling container [27]. These collisions result in the deformation, fracturing, and welding of the particles, leading to a reduction in particle size and an increase in surface area (Figure 1a–c) [28]. The creation of fresh surfaces during the milling process exposes highly reactive sites that can enhance the hydrogen absorption and desorption kinetics. The introduction of defects, such as dislocations and grain boundaries, can provide pathways for hydrogen diffusion and facilitate the nucleation of hydride phases [29]. Additionally, in controlled reaction mechanical milling, the application of external disturbances, such as adjusting the grinding forces to high-energy shearing (HES, Figure 1d) and intense impacts (IMP, Figure 1e), can enhance the exposure of the active surfaces of the grinding particles [30]. The HES mode utilizes vibrational or shaking actions to accelerate the milling media, thereby inducing high-energy collisions within the milling jar. This process effectively converts mechanical energy into plastic deformation and grain refinement of the materials, consequently reducing the milling duration. However, continuous high-frequency vibration may cause the temperature of the materials and the milling jar to rise, potentially affecting the physical properties of the materials. Additionally, some energy may be dissipated through vibrations and sound, resulting in reduced energy efficiency. In the IMP mode, the milling media leverage the force generated during rotational motion to crush the materials. The IMP mode exhibits a strong crushing capability, making it suitable for pulverizing materials of high hardness and strength. By adjusting the milling speed and duration, the particle size of the materials can be precisely controlled, making it appropriate for the preparation of nano-materials with varied particle sizes. However, it is important to note that high-speed impacts may introduce impurities from the milling media, affecting the purity of the materials.

#### 3.1.2. Optimization of Milling Parameters

The hydrogen storage performance of ball-milled magnesium-based materials is strongly influenced by the milling parameters, such as the milling time, milling speed, ball-to-powder ratio, and milling atmosphere [31]. Optimizing these parameters is crucial for achieving the desired particle size, microstructure, and hydrogen storage properties.

Milling time: Increasing the milling time generally leads to a reduction in particle size and an increase in surface area, which can improve the kinetic properties of magnesium-based materials. However, prolonged milling can also result in the agglomeration of particles and the formation of stable oxide layers, which can hinder hydrogen absorption and desorption [32]. Optimizing the milling time is essential to achieve a balance between particle size reduction and the prevention of deleterious effects. Fátay et al. [33] investigated the effect of milling time on the dehydrogenation performance of MgH_2_. They found that as the milling time increased from 1 h to 20 h, the particle size of MgH_2_ decreased from 900 nm to about 13 nm, and the specific surface area increased from 1.6 m^2^/g to 33.2 m^2^/g. When the milling time was 20 h, the dehydrogenation temperature of MgH_2_ decreased from 410 °C to 335 °C, and the activation energy decreased from 162 kJ/mol to 94 kJ/mol, indicating significantly improved kinetic properties.

Milling speed: The milling speed determines the energy input during the ball-milling process. Higher milling speeds generally result in more intense collisions and a greater degree of particle size reduction. However, excessively high milling speeds can lead to increased temperature and the formation of stable phases, which may negatively impact the hydrogen storage properties [34]. Optimizing the milling speed is necessary to achieve efficient particle size reduction while minimizing undesirable phase transformations. Révész et al. [35] studied the influence of milling speed on the microstructure and hydrogen sorption properties of ball-milled MgH_2_. They observed that increasing the milling speed from 200 rpm to 600 rpm led to a decrease in crystallite size from 18 nm to 7 nm and an increase in the specific surface area from 11 m^2^/g to 60 m^2^/g. As a result, the dehydrogenation temperature of MgH_2_ decreased from 400 °C to 350 °C, and the activation energy decreased from 120 kJ/mol to 90 kJ/mol, demonstrating enhanced kinetic properties.

Ball-to-powder ratio: The ball-to-powder ratio (BPR) is defined as the mass ratio of the milling balls to the powder sample. A higher BPR generally results in a greater energy input and more efficient particle size reduction. However, an excessively high BPR can lead to increased contamination from the milling tools and the formation of stable phases [36]. Optimizing the BPR is important to achieve effective particle size reduction while minimizing contamination and preserving the desired phase composition.

Milling atmosphere: The milling atmosphere can significantly influence the hydrogen storage properties of magnesium-based materials. Milling under a hydrogen atmosphere can promote the in situ formation of hydride phases and enhance the hydrogen absorption kinetics [37]. Milling under an inert atmosphere, such as argon or nitrogen, can prevent oxidation and minimize contamination during the milling process. The choice of milling atmosphere depends on the specific requirements of the material and the desired hydrogen storage properties.

#### 3.1.3. Modification Strategies

Various modification strategies have been employed to enhance the hydrogen storage properties of ball-milled magnesium-based materials, such as the addition of catalytic additives, the incorporation of nanostructured carbon materials, and the application of reactive ball milling [38].

Catalytic additives: The addition of transition metal catalysts, such as Ni, Ti, Fe, and Co, can significantly enhance the hydrogen absorption and desorption kinetics of magnesium-based materials. These catalysts promote the dissociation of hydrogen molecules and the recombination of hydrogen atoms, lowering the activation energy barriers for hydrogen sorption [39]. The catalytic effect is attributed to the spillover mechanism, where hydrogen molecules are dissociated on the catalyst surface and the resulting hydrogen atoms migrate to the magnesium matrix, facilitating the formation of hydride phases [40]. Zhou et al. [41] prepared nanocrystalline MgH_2_ with an average particle size of 7 nm by ball milling MgH_2_ with 5 wt.% TiF_3_ catalyst. The resulting material exhibited a high hydrogen storage capacity of 6.8 wt.% at 300 °C, with fast absorption and desorption kinetics. The enhanced performance was attributed to the small particle size, high surface area, and the catalytic effect of TiF_3_. Molinas et al. [42] demonstrated the scalability of the ball-milling method for the production of MgH_2_-based hydrogen storage materials. They used an attritor-type ball mill with a capacity of 0.5–1 kg to prepare MgH_2_ with 10 wt.% Zr-Ni alloy as a catalyst. The resulting material exhibited a hydrogen storage capacity between 5.3 and 5.6 wt.% and fast absorption/desorption kinetics at around 300 °C, with 90% of the total hydrogen absorbed in less than 100 s and desorbed in less than 300 s. These results were comparable to those obtained with materials prepared using a small laboratory mill, highlighting the potential of the ball-milling method for semi-industrial scale production of magnesium-based hydrogen storage materials.

Nanostructured carbon materials: The incorporation of nanostructured carbon materials, such as carbon nanotubes (CNTs), graphene, and carbon nanofibers, can improve the hydrogen storage properties of magnesium-based materials. These carbon materials act as structural supports, preventing particle agglomeration and maintaining a high surface area during repeated absorption–desorption cycles [43]. Additionally, the high thermal conductivity of carbon materials can enhance the heat transfer properties of the system, facilitating the heat management during hydrogen sorption processes [44].

Reactive ball milling: Reactive ball milling involves the milling of magnesium with reactive gases, such as hydrogen or nitrogen, to promote the in situ formation of hydride or nitride phases. This technique can lead to the formation of nanostructured and metastable phases with enhanced hydrogen storage properties [45]. Reactive ball milling can also improve the dispersion of catalytic additives and create a more homogeneous distribution of the reactive phases within the magnesium matrix [46]. Shao et al. [47] applied reactive ball milling to prepare a magnesium–nitrogen composite and investigated its hydrogen storage properties. The in situ formation of magnesium nitride during the milling process led to a significant improvement in the hydrogen absorption and desorption kinetics compared to pure magnesium. The magnesium–nitrogen composite exhibited a hydrogen desorption capacity of 5.8 wt.% at 275 °C, with fast kinetics and good cycling stability.

### 3.2. Methanol-Wrapped Chemical Vapor Deposition (MWCVD)

#### 3.2.1. Principles and Processes

The methanol-wrapped chemical vapor deposition (MWCVD) method is a novel technique for the preparation of magnesium-based hydrogen storage materials with controlled morphology and composition. This method involves the evaporation of magnesium in a methanol atmosphere, leading to the formation of methanol-wrapped magnesium vapor [48]. The vapor is then deposited onto a substrate, where it undergoes a self-assembly process to form nanostructured magnesium-based materials.

**Figure 2:**
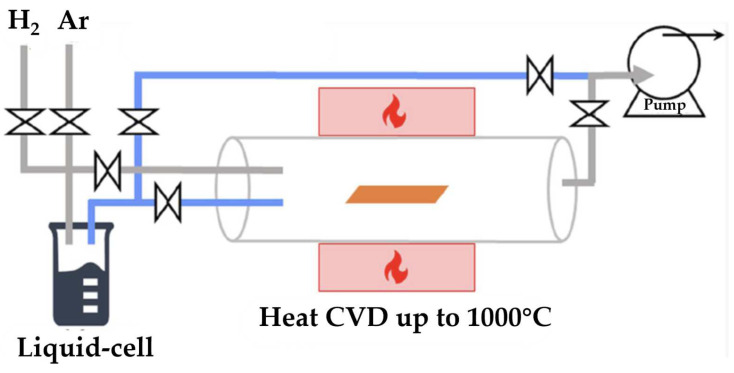
Schematic illustration of the MWCVD process [49].

As shown in Figure 2, the MWCVD process can be divided into three main steps: (1) evaporation of magnesium in a methanol atmosphere, (2) transport of the methanol-wrapped magnesium vapor to the substrate, and (3) deposition and self-assembly of the magnesium-based nanostructures on the substrate [49,50]. The methanol atmosphere plays a crucial role in the formation of the nanostructures, as it acts as a surface-active agent and a structure-directing agent during the deposition process.

The evaporation of magnesium in the methanol atmosphere leads to the formation of magnesium–methanol complexes, which have a lower evaporation temperature compared to pure magnesium. This allows for the formation of a high-density magnesium vapor at relatively low temperatures, facilitating the efficient transport of the vapor to the substrate. The methanol molecules surrounding the magnesium atoms in the vapor phase also help to prevent the agglomeration and oxidation of the magnesium during the transport process.

Upon reaching the substrate, the methanol-wrapped magnesium vapor undergoes a self-assembly process, leading to the formation of various nanostructures, such as nanoparticles, nanowires, and nanosheets [51]. The morphology and size of the nanostructures can be controlled by adjusting the deposition parameters, such as the substrate temperature, methanol flow rate, and deposition time. The methanol molecules adsorbed on the surface of the nanostructures act as a protective layer, preventing oxidation and facilitating the hydrogen absorption and desorption processes [52]. Several studies have reported the successful preparation of magnesium-based hydrogen storage materials using the MWCVD method. 

#### 3.2.2. Influence of Process Parameters

The hydrogen storage properties of magnesium-based materials prepared by the MWCVD method are strongly influenced by the process parameters, such as the deposition temperature, methanol flow rate, and substrate type.

Deposition temperature: The deposition temperature plays a crucial role in determining the morphology and size of the magnesium-based nanostructures. Higher deposition temperatures generally lead to the formation of larger nanostructures with a lower surface area, while lower temperatures favor the formation of smaller nanostructures with a higher surface area [53]. Optimizing the deposition temperature is essential to achieve the desired morphology and hydrogen storage properties. Zhu et al. [54] studied the effect of deposition temperature on the morphology of Mg nanowires prepared by MWCVD. They observed that increasing the deposition temperature from 200 °C to 400 °C led to a decrease in the aspect ratio of the nanowires from 100 to 20 and an increase in the average diameter from 30 nm to 150 nm. The higher deposition temperature resulted in a higher growth rate and a more rapid thickening of the nanowires.

Methanol flow rate: The methanol flow rate affects the concentration of methanol in the vapor phase and the rate of magnesium deposition. Higher methanol flow rates lead to a higher concentration of methanol in the vapor phase, which can enhance the surface protection and structure-directing effects of methanol [55]. However, excessively high methanol flow rates may result in the formation of a thick methanol layer on the substrate, hindering the deposition and self-assembly of the magnesium-based nanostructures. Optimizing the methanol flow rate is necessary to achieve a balance between the surface protection and the efficient deposition of the nanostructures.

Substrate type: The type of substrate used in the MWCVD process can significantly influence the morphology and orientation of the deposited magnesium-based nanostructures. Different substrates have different surface energies, crystallographic orientations, and chemical properties, which can affect the nucleation and growth of the nanostructures [56]. Commonly used substrates for the MWCVD of magnesium-based materials include silicon, glass, and metal foils. The choice of substrate depends on the desired morphology, orientation, and application of the nanostructures.

#### 3.2.3. Selection of Catalysts

The incorporation of catalytic additives during the MWCVD process can further enhance the hydrogen storage properties of magnesium-based materials. Transition metal catalysts, such as Ni, Ti, and Fe, are commonly used to improve the hydrogen absorption and desorption kinetics [57]. These catalysts can be co-deposited with magnesium during the MWCVD process, forming nanocomposites with enhanced catalytic activity.

The selection of appropriate catalysts and their optimization are crucial for achieving high hydrogen storage performance. Factors to consider when selecting catalysts include their catalytic activity, selectivity, stability, and compatibility with the magnesium-based matrix [58]. The concentration and distribution of the catalysts in the nanocomposites also play a significant role in determining the hydrogen storage properties. Xie et al. [59] investigated the catalytic effect of Ni nanoparticles on the desorption kinetics of MgH_2_ nanowires prepared by MWCVD. They found that the addition of 5 wt.% Ni nanoparticles reduced the desorption temperature from 350 °C to 250 °C and increased the desorption rate by a factor of 5 compared to pure MgH_2_ nanowires. The enhanced performance was attributed to the catalytic effect of Ni nanoparticles, which facilitated the dissociation of H_2_ molecules and the recombination of H atoms.

### 3.3. Plasma-Assisted Ball Milling

#### 3.3.1. Plasma Activation Mechanism

Plasma-assisted ball milling is an advanced technique that combines the advantages of mechanical ball milling and plasma processing for the preparation of magnesium-based hydrogen storage materials. The plasma activation mechanism involves the generation of highly reactive species, such as ions, electrons, and radicals, which can interact with the surface of the magnesium particles during the ball-milling process [60].

The plasma species can induce various surface modifications on the magnesium particles, such as surface cleaning, surface activation, and surface functionalization [61]. The high-energy plasma species can remove the surface oxide layers and other contaminants from the magnesium particles, creating fresh and highly reactive surfaces. The plasma activation can also create surface defects, such as vacancies and dislocations, which can act as active sites for hydrogen adsorption and dissociation [62]. Gennari et al. [63] investigated the effect of plasma-assisted ball milling on the hydrogen storage properties of Mg-Ti composites. They found that plasma treatment during the milling process led to a significant reduction in the desorption temperature (from 350 °C to 250 °C) and an increase in the hydrogen desorption rate by a factor of 3 compared to conventional ball milling. The enhanced performance was attributed to the formation of a highly reactive and defect-rich surface layer induced by the plasma treatment and the catalytic effect of the Ti particles.

**Figure 3:**
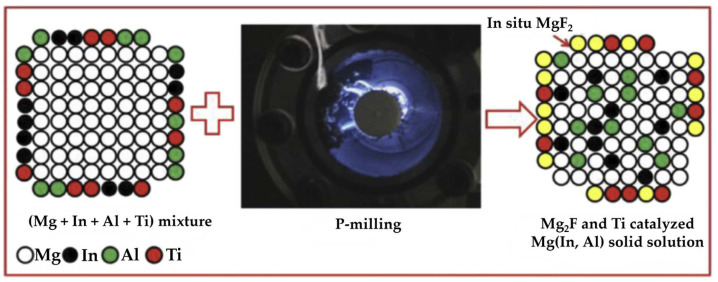
Schematic illustration of the preparation process of Mg_85_In_5_Al_5_Ti_5_ alloy by P-milling [65].

Moreover, the plasma species can promote the formation of metastable phases and nanostructures on the surface of the magnesium particles [64]. The rapid heating and cooling induced by the plasma can lead to the formation of non-equilibrium phases and nanocrystalline structures, which can enhance the hydrogen storage properties of the magnesium-based materials. As shown in Figure 3, MgF_2_ and Ti-catalyzed Mg(In, Al) were in situ generated under the continuous impact of ball milling and the rapid heating and melting effect of the aforementioned plasma discharge [65].

Several studies have demonstrated the effectiveness of plasma-assisted ball milling in improving the hydrogen storage properties of magnesium-based materials. The plasma treatment led to the formation of a nanocrystalline structure and a uniform distribution of nickel nanoparticles on the surface of the magnesium particles. The nanocomposite exhibited a hydrogen storage capacity of 6.2 wt.% at 300 °C, with fast absorption and desorption kinetics. The enhanced performance was attributed to the synergistic effect of the nanocrystalline structure, the catalytic effect of nickel, and the surface activation induced by the plasma treatment.

The research cases highlight the effectiveness of plasma-assisted ball milling in preparing magnesium-based hydrogen storage materials with enhanced hydrogen storage properties. The plasma activation mechanism, combined with the optimization of ball-milling parameters, can lead to significant improvements in the surface properties, microstructure, and hydrogen storage performance of these materials.

#### 3.3.2. Optimization of Ball-Milling Parameters

The hydrogen storage performance of plasma-assisted ball-milled magnesium-based materials is influenced by both the plasma processing parameters and the ball-milling parameters. The optimization of these parameters is crucial for achieving the desired surface modifications and hydrogen storage properties. Table 2 summarized key parameters in plasma-assisted ball milling.

Plasma processing parameters: The plasma processing parameters, such as the plasma power, gas composition, and treatment time, should be optimized to achieve the desired surface modifications and activation effects [69]. Higher plasma powers generally lead to more intense surface modifications, but excessive power may cause surface damage and deteriorate the hydrogen storage properties. The gas composition of the plasma, such as the ratio of hydrogen to argon, can influence the type and extent of surface modifications. Longer treatment times can result in more extensive surface modifications, but prolonged exposure to plasma may lead to surface saturation and a decrease in the hydrogen storage capacity.

Ball-milling parameters: The ball-milling parameters, such as the milling time, milling speed, and ball-to-powder ratio, should be adjusted to obtain the optimal particle size, surface area, and microstructure of the magnesium-based materials [70]. Longer milling times can lead to a greater reduction in particle size and an increase in surface area, but excessive milling may cause particle agglomeration and a decrease in the hydrogen storage capacity. Higher milling speeds can result in more intense particle deformation and surface modifications, but excessive speeds may lead to increased temperature and the formation of stable phases. Cao et al. [65] investigated the effect of milling speed on the thermodynamics and kinetics of Mg_85_In_5_Al_5_Ti_5_ alloy synthesized by plasma milling. They found that increasing the milling speed from 200 rpm to 400 rpm led to a decrease in the desorption temperature from 340 °C to 275 °C and an increase in the hydrogen desorption capacity from 5.2 wt.% to 6.0 wt.%. The improved performance was attributed to the enhanced surface activation and defect generation induced by the higher milling speed. A higher ball-to-powder ratio can lead to more efficient particle size reduction and surface modification, but an excessively high ratio may cause contamination from the milling tools.

Optimization of the plasma processing and ball-milling parameters requires a systematic investigation of their effects on the surface properties, microstructure, and hydrogen storage performance of the magnesium-based materials. Design of experiments, such as factorial design and response surface methodology, can be employed to identify the optimal parameter combinations for achieving the desired hydrogen storage properties [71].

### 3.4. Organic Ligand-Assisted Method

**Figure 4:**
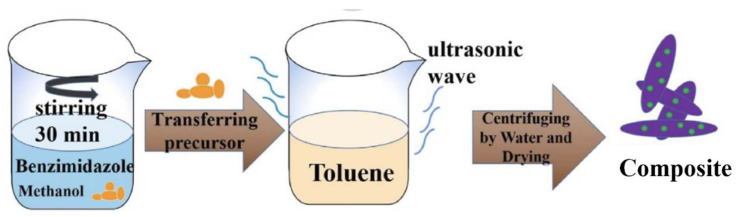
Synthesis procedure of composite through organic ligand-assisted strategy [72].

Organic ligand-assisted synthesis is a promising method for the preparation of magnesium-based hydrogen storage materials with tailored nanostructures and surface properties. Various organic ligands, such as surfactants, polymers, and organic acids, can be used to control the nucleation, growth, and assembly of magnesium nanoparticles during the synthesis process (Figure 4) [72].

Several studies have reported the successful preparation of magnesium-based hydrogen storage materials using the organic ligand-assisted method. For example, Grignard reagents provide a simple pathway for the thermal decomposition of Mg and/or MgH_2_ [73]. For instance, di-tert-butyl magnesium decomposes to form MgH_2_/Mg at a low temperature of 167 °C, which is 100 °C lower than the temperature required for di-n-butyl magnesium to be converted to MgH_2_. Additionally, the formation of metastable gamma-MgH_2_ enables the MgH_2_ synthesized from di-tert-butyl magnesium precursor to release hydrogen at 100 °C [73]. Aguey-Zinsou et al. [74] synthesized MgH_2_ nanoparticles with a core-shell structure using an organic ligand-assisted method. They used oleic acid as a capping agent to control the growth and prevent the agglomeration of the nanoparticles. The resulting core-shell nanoparticles exhibited a high hydrogen storage capacity (6.8 wt.%) and fast absorption/desorption kinetics at 200 °C, which was attributed to the unique nanostructure and the protective oleic acid layer.

These research cases demonstrate the effectiveness of the organic ligand-assisted method in preparing magnesium-based hydrogen storage materials with tailored nanostructures and enhanced hydrogen storage properties. The choice of appropriate organic ligands and the optimization of the synthesis conditions are crucial for achieving the desired nanostructure and hydrogen storage performance.

#### 3.4.1. Roles of Organic Ligands

Surfactants: Surfactants are amphiphilic molecules that consist of a hydrophilic head and a hydrophobic tail. They can adsorb on the surface of magnesium nanoparticles and form a protective layer, preventing particle agglomeration and controlling the growth of the nanoparticles [75]. Commonly used surfactants for the synthesis of magnesium-based materials include oleylamine, oleic acid, and cetyltrimethylammonium bromide (CTAB). These surfactants can also act as structure-directing agents, guiding the assembly of the nanoparticles into specific morphologies, such as nanorods, nanowires, and nanosheets [76]. Xia et al. [76] compared the hydrogen storage properties of MgH_2_ nanoparticles, nanowires, and nanosheets prepared by different methods. They found that the nanosheets exhibited the highest hydrogen storage capacity (7.2 wt.%) and the fastest absorption/desorption kinetics among the three nanostructures. The superior performance of the nanosheets was attributed to their high surface area, short hydrogen diffusion paths, and the presence of numerous surface defects and active sites.

Polymers: Polymers can serve as stabilizing agents and structure-directing agents in the synthesis of magnesium-based hydrogen storage materials. They can bind to the surface of the magnesium nanoparticles through functional groups, such as carboxyl, hydroxyl, and amino groups, forming a protective shell [77]. The polymer shell can prevent particle agglomeration, control the particle size and morphology, and improve the dispersibility of the nanoparticles. Commonly used polymers include polyvinylpyrrolidone (PVP), polyethylene glycol (PEG), and polyacrylic acid (PAA).

Organic acids: Organic acids, such as acetic acid, oxalic acid, and citric acid, can act as chelating agents and reducing agents in the synthesis of magnesium-based hydrogen storage materials. They can form complexes with magnesium ions, controlling the nucleation and growth of the nanoparticles [78]. The organic acids can also serve as a source of carbon, which can improve the thermal conductivity and mechanical properties of the magnesium-based materials [79].

The choice of organic ligands depends on the desired nanostructure, surface properties, and hydrogen storage performance of the magnesium-based materials. The concentration and ratio of the organic ligands can be adjusted to optimize the size, morphology, and surface chemistry of the nanoparticles [80].

#### 3.4.2. Preparation Process and Mechanism

The organic ligand-assisted synthesis of magnesium-based hydrogen storage materials typically involves the reduction of magnesium salts in the presence of organic ligands under controlled conditions. The reduction process can be carried out using various reducing agents, such as sodium borohydride, lithium aluminum hydride, or hydrogen gas [81]. Table 3 summarizes the effects of different organic ligands used in the synthesis of magnesium-based hydrogen storage materials.

The general preparation process is shown in Figure 4. Firstly, magnesium salt precursor and organic ligands are dissolved in a suitable solvent, such as ethanol, methanol, or tetrahydrofuran (THF), to form a homogeneous solution. Then, a reducing agent is added to the solution, initiating the reduction of the magnesium ions to magnesium nanoparticles. The organic ligands adsorb on the surface of the newly formed magnesium nanoparticles, preventing their agglomeration and controlling their growth. The nanoparticles are allowed to grow and assemble into the desired nanostructure under controlled conditions, such as temperature, pH, and stirring rate. The resulting nanostructured magnesium-based material is collected by centrifugation or filtration, washed with solvents to remove excess ligands, and dried under vacuum. The mechanism of organic ligand-assisted synthesis involves the interplay between the reduction of magnesium ions, the adsorption of organic ligands, and the growth and assembly of the nanoparticles [86]. The organic ligands can influence the reduction kinetics by complexing with the magnesium ions, affecting the nucleation rate and the size of the nanoparticles. The adsorption of the ligands on the nanoparticle surface can lower the surface energy, preventing particle agglomeration and stabilizing the nanostructure [87]. The ligands can also guide the growth and assembly of the nanoparticles through selective adsorption on specific crystal faces, leading to the formation of anisotropic nanostructures.

### 3.5. Other Emerging Methods

#### 3.5.1. Rapid Solid-Phase Reaction

The rapid solid-phase reaction method is a novel technique for the preparation of magnesium-based hydrogen storage materials with high purity and uniform composition. This method involves the high-energy milling of magnesium with other solid reactants, such as metal hydrides or metal oxides, under a hydrogen atmosphere [88].

The high-energy milling process induces mechano-chemical reactions between the solid reactants, leading to the formation of magnesium-based hydrides or composites. The rapid solid-phase reactions are driven by the high mechanical energy input and the increased surface area and reactivity of the milled particles [89]. The hydrogen atmosphere during the milling process helps to maintain a reducing environment and prevents the oxidation of the magnesium-based materials.

One of the advantages of the rapid solid-phase reaction method is the ability to produce high-purity magnesium-based materials with uniform composition [90]. The solid-state reactions between the reactants ensure a homogeneous distribution of the components in the final product. Additionally, the high-energy milling process can lead to the formation of nanocrystalline or amorphous structures, which can enhance the hydrogen storage properties of the materials [91].

For example, Shao et al. [92] prepared a magnesium-nickel hydride composite using the rapid solid-phase reaction method. Magnesium and nickel hydride powders were milled under a hydrogen atmosphere for 10 h, resulting in the formation of a nanocrystalline MgH_2_-Ni composite. The composite exhibited a hydrogen storage capacity of 6.5 wt.% at 300 °C, with fast absorption and desorption kinetics. The enhanced performance was attributed to the uniform distribution of the nickel catalyst and the nanocrystalline structure of the magnesium hydride matrix.

#### 3.5.2. Mechanical-Induced Reaction

The mechanical-induced reaction method is a mechanochemical technique that utilizes the high shear forces generated during the extrusion or milling process to promote solid-state reactions between magnesium and other components. The high shear forces can break down the oxide layers on the surface of the magnesium particles, create fresh surfaces, and enhance the contact and diffusion between the reactants [93].

The mechanical-induced reaction method has been used to prepare magnesium-based hydrides and composites with improved hydrogen storage properties. Mechanical milling induced twinning deformations in MgH_2_, further promoting its thermodynamic instability. Non-in situ results indicate that magnesium metal crystallizes non-uniformly on large hydride particles. Smaller particles either fully converted to magnesium or remain completely in the hydride state. This phenomenon may be due to changes in the crystallography of the particle surface, variations in the hydride structure on the surface, alterations in defect content, or the proximity of residual magnesium hydride phases [94].

El-Eskandarany et al. [95] obtained a new metastable fcc-MgH_2_ nanocrystalline phase during the mechanically induced plastic deformation process. Their findings indicate that 200 passes of cold rolling on MgH_2_ powder resulted in severe plastic deformation and the formation of a micro-lathe composed of gamma- and β-MgH_2_ phases. The cold-rolled powder was characterized by various types of defects such as dislocations, stacking faults, and twins during high-energy ball milling. Extended ball milling (50 h) destabilized the beta-MgH_2_ (most stable phase) and γ-MgH_2_ (metastable phase) to form a new face-centered cubic (FCC) phase. This phase exhibited a higher hydrogen storage capacity (6.6 wt.%) and released hydrogen within 7 min at 275 °C.

#### 3.5.3. Electrochemically Assisted Synthesis

The electrochemically assisted synthesis method is a novel approach for the preparation of magnesium-based hydrogen storage materials using electrochemical techniques. This method involves the electrodeposition of magnesium or magnesium alloys onto a substrate from an electrolyte solution, followed by hydrogen absorption and desorption cycles [96].

The electrochemical synthesis allows for the control of the composition, morphology, and thickness of the magnesium-based materials by adjusting the electrodeposition parameters, such as the current density, deposition time, and electrolyte composition [97]. The electrodeposited magnesium-based materials often exhibit nanocrystalline or amorphous structures, high surface areas, and improved hydrogen storage properties compared to bulk materials.

One of the advantages of the electrochemically assisted synthesis method is the ability to prepare thin films or coatings of magnesium-based materials on various substrates, such as metal foils, carbon materials, or metal-organic frameworks [98]. The thin film geometry can facilitate the hydrogen diffusion and improve the kinetics of hydrogen absorption and desorption.

Knotek et al. [99] prepared hydrides based on Mg_2_Ni and Mg_12_RE using electrochemical hydrogenation. Their results indicate that while both Mg_2_Ni and Mg_12_RE phases support hydrogen absorption, they do so in different ways. The Mg_2_Ni phase maximizes hydrogen concentration beneath the binary alloy surface, whereas the Mg_12_RE phase reduces the maximum surface concentration of hydrogen but facilitates its diffusion inward. The influence of the Mg_12_RE phase on hydrogen diffusion can reduce the decomposition temperature of MgH_2_ by more than 190 °C.

These emerging methods offer new opportunities for the preparation of magnesium-based hydrogen storage materials with unique nanostructures, compositions, and properties. The rapid solid-phase reaction, shear-induced reaction, and electrochemically assisted synthesis methods can produce high-purity, uniform, and nanostructured materials with enhanced hydrogen storage performance. Further research and development of these methods may lead to the discovery of novel magnesium-based materials with superior hydrogen storage properties.

## 4. Comparison and Evaluation of Preparation Methods

### 4.1. Analysis of Different Preparation Methods

Each preparation method for magnesium-based hydrogen storage materials has its own advantages and disadvantages, which are summarized in Table 4.

The choice of the preparation method depends on the specific requirements of the hydrogen storage application, such as the desired hydrogen storage capacity, kinetics, and cycling stability, as well as the scalability and cost-effectiveness of the process.

The mechanical ball-milling method is widely used due to its simplicity, versatility, and scalability. It can produce nanostructured materials with high surface areas and defect densities, which can enhance the hydrogen storage properties. However, the long milling times and the potential contamination from the milling tools are some of the drawbacks of this method.

The methanol-wrapped chemical vapor deposition (MWCVD) method can produce magnesium-based materials with controlled morphology and composition, such as nanowires, nanorods, and nanocomposites. The methanol atmosphere during the deposition process can prevent oxidation and facilitate the formation of unique nanostructures. However, the high temperature and vacuum conditions required for the MWCVD process may limit its scalability and cost-effectiveness.

The plasma-assisted ball-milling method combines the advantages of ball milling and plasma processing, leading to enhanced surface modification and activation of the magnesium-based materials. The plasma treatment can create highly reactive surfaces, defects, and metastable phases, which can improve the hydrogen storage kinetics. However, the complexity of the plasma setup and the optimization of the plasma parameters may be challenging.

The organic ligand-assisted synthesis method can produce magnesium-based materials with tailored nanostructures and surface properties, such as nanoparticles, nanorods, and core-shell structures. The organic ligands can control the nucleation, growth, and assembly of the nanostructures, leading to improved dispersibility and stability. However, the removal of the organic ligands after the synthesis may be difficult and may affect the hydrogen storage performance.

The rapid solid-phase reaction, shear-induced reaction, and electrochemically assisted synthesis methods are emerging techniques that can produce high-purity and uniform magnesium-based materials with unique nanostructures and properties. These methods offer new possibilities for the preparation of advanced magnesium-based hydrogen storage materials. However, their scalability, cost-effectiveness, and reproducibility need to be further investigated.

### 4.2. Influence of Preparation Methods on Hydrogen Storage Properties

The preparation methods have a significant influence on the hydrogen storage properties of magnesium-based materials, such as the hydrogen storage capacity, kinetics, and cycling stability. The nanostructure, surface properties, and composition of the materials, which are largely determined by the preparation methods, play crucial roles in their hydrogen storage performance.

The preparation methods that can produce materials with small particle sizes, high surface areas, and defect densities, such as ball milling and plasma-assisted ball milling, can enhance the hydrogen absorption and desorption kinetics. The small particle sizes and high surface areas provide more sites for hydrogen dissociation and recombination, while the defects and strain induced by the milling process can facilitate hydrogen diffusion [104]. Noticed, the key breakthrough of this method lies in addressing the decline in hydrogen storage capacity caused by the introduction of surface oxidation and impurities during the milling process.

The preparation methods that can control the morphology and composition of the materials, such as MWCVD and organic ligand-assisted synthesis, can lead to improved hydrogen storage properties. The unique nanostructures, such as nanowires and nanorods, can provide short hydrogen diffusion paths and high surface areas, leading to fast kinetics and high storage capacities [105]. The incorporation of catalytic additives or the formation of nanocomposites can further enhance the hydrogen storage performance by promoting hydrogen dissociation and recombination, and by preventing particle agglomeration.

The preparation methods that can produce high-purity and uniform materials, such as rapid solid-phase reaction and electrochemically assisted synthesis, can lead to improved hydrogen storage properties. The high purity and uniformity of the materials can minimize the negative effects of impurities and ensure consistent hydrogen storage performance [106]. The nanocrystalline or amorphous structures produced by these methods can also enhance the hydrogen storage kinetics and capacity.

In summary, the preparation methods that can produce magnesium-based materials with optimized nanostructures, surface properties, and compositions are essential for achieving high hydrogen storage performance. The selection of the appropriate preparation method depends on the specific requirements of the hydrogen storage application, such as the desired storage capacity, kinetics, and cycling stability.

### 4.3. Assessment of Practical Application Potential

The practical application potential of magnesium-based hydrogen storage materials prepared by different methods depends on various factors, such as the hydrogen storage performance, scalability, cost-effectiveness, and safety.

The mechanical ball-milling method has been widely studied and has shown promising results for practical applications. The ball-milled magnesium-based materials have demonstrated high hydrogen storage capacities (up to 7 wt.%), fast kinetics, and good cycling stability [107]. The scalability and cost-effectiveness of the ball-milling process are also favorable for industrial-scale production. However, the long milling times and the potential contamination issues need to be addressed for practical applications.

The MWCVD method has shown potential for the production of high-performance magnesium-based hydrogen storage materials with controlled nanostructures and compositions. The MWCVD-prepared materials have exhibited high hydrogen storage capacities (up to 7 wt.%) and fast kinetics [108]. However, the scalability and cost-effectiveness of the MWCVD process may be limited by the high temperature and vacuum conditions required for the deposition process.

The plasma-assisted ball-milling method has demonstrated improved hydrogen storage performance compared to the conventional ball-milling method. The plasma-assisted ball-milled materials have shown high hydrogen storage capacities (up to 6.5 wt.%), fast kinetics, and good cycling stability [109]. The scalability of the plasma-assisted ball-milling process is similar to that of the conventional ball-milling process, but the additional cost and complexity of the plasma setup need to be considered.

The organic ligand-assisted synthesis method has shown promising results for the production of magnesium-based materials with tailored nanostructures and surface properties. The materials prepared by this method have exhibited high hydrogen storage capacities (up to 7 wt.%) and fast kinetics. However, the scalability and cost-effectiveness of the organic ligand-assisted synthesis method may be limited by the multi-step synthesis process and the need for the removal of the organic ligands.

The rapid solid-phase reaction, shear-induced reaction, and electrochemically assisted synthesis methods are emerging techniques that have shown potential for the production of high-performance magnesium-based hydrogen storage materials. These methods can produce high-purity and uniform materials with unique nanostructures and properties. However, their practical application potential needs to be further evaluated in terms of scalability, cost-effectiveness, and safety.

## 5. Summary and Outlook

### 5.1. Summary of Research Status on Preparation Methods

This review has provided a comprehensive overview of the recent advances in the preparation methods of magnesium-based hydrogen storage materials, including mechanical ball milling, methanol-wrapped chemical vapor deposition, plasma-assisted ball milling, organic ligand-assisted synthesis, and other emerging methods. Each method has its own advantages and disadvantages in terms of the control over the nanostructure, composition, and surface properties of the materials, as well as the scalability and cost-effectiveness of the process.

The practical application potential of magnesium-based hydrogen storage materials prepared by different methods varies depending on their hydrogen storage performance, scalability, cost-effectiveness, and safety. The mechanical ball-milling and plasma-assisted ball-milling methods have shown the most promise for practical applications due to their scalability and cost-effectiveness. The MWCVD and organic ligand-assisted synthesis methods have shown potential for the production of high-performance materials, but their scalability and cost-effectiveness need to be improved. The emerging methods, such as rapid solid-phase reaction, shear-induced reaction, and electrochemically assisted synthesis, require further research and development to assess their practical application potential.

### 5.2. Existing Problems and Challenges

1. Hydrogen Storage Capacity and Kinetics: The theoretical hydrogen storage capacity of magnesium-based materials is 7.6 wt.% (MgH_2_). However, due to the presence of impurities, surface oxidation, and incomplete hydrogenation, the practically achievable capacity often falls short of this ideal value. To meet the U.S. Department of Energy’s target of 6.5 wt.% for light-duty vehicles, further improvements in the hydrogen storage capacity of magnesium-based materials are necessary. Additionally, the hydrogen absorption and desorption kinetics of these materials exhibit significant limitations, hindering their ability to achieve rapid hydrogen uptake and release at moderate temperatures. The high activation energy barriers for hydrogen dissociation and recombination, coupled with the slow diffusion of hydrogen in the solid state, are the primary bottlenecks restricting the rates of hydrogenation and dehydrogenation. Consequently, research efforts are currently focused on reducing these energy barriers and enhancing hydrogen diffusion rates, which are key concerns for both the academic and industrial communities.
2. Cycling Stability: The cycling stability and reversibility of magnesium-based materials need substantial enhancement to ensure their reliable performance in long-term practical applications. During multiple hydrogen absorption–desorption cycles, the hydrogen storage capacity and kinetics of these materials tend to degrade gradually. This degradation is typically caused by factors such as particle agglomeration, surface oxidation, and phase segregation. Particle agglomeration leads to a reduction in the specific surface area, thereby decreasing the material’s reactivity. Surface oxidation forms an oxide layer that hinders hydrogen absorption and desorption processes, while phase segregation results in structural heterogeneity within the material, further impairing its performance. Thus, effectively mitigating these degradation mechanisms and improving the cycling stability of magnesium-based materials are critical areas of future research.
3. Cost and Safety: Although some advanced preparation methods have been demonstrated to significantly enhance the performance of magnesium-based hydrogen storage materials, their scalability and cost-effectiveness for industrial-scale production require further optimization. These methods often involve high temperatures, vacuum conditions, and complex multi-step synthesis processes, thereby increasing production costs and technical challenges. Additionally, the safety aspects of magnesium-based hydrogen storage materials cannot be overlooked. Magnesium is a highly reactive metal, and its hydrides are prone to oxidation and ignition upon exposure to air or moisture. Therefore, developing effective surface protection strategies and safe handling protocols is essential to ensure the safety and reliability of magnesium-based materials in practical applications. These improvements will facilitate the advancement of magnesium-based hydrogen storage materials towards practical use, enhancing their competitiveness in the market.

### 5.3. Future Research Directions

Based on the current research status and challenges, the future research directions and development trends in the preparation of magnesium-based hydrogen storage materials may include are summarized in Table 5.

Based on the content summarized in Table 5, we believe the following are some focal points for future research on the preparation methods of magnesium-based hydrogen storage materials.

Development of novel catalytic additives and nanostructured supports to further enhance the hydrogen storage kinetics and capacity of magnesium-based materials. The exploration of new catalytic systems, such as bimetallic alloys, metal oxides, and metal-organic frameworks, and the optimization of their composition, size, and distribution, can lead to improved hydrogen storage performance.

Rational design and synthesis of magnesium-based nanostructures with optimized morphology, size, and surface properties for enhanced hydrogen storage. The use of advanced characterization techniques, such as in situ X-ray diffraction, neutron scattering, and transmission electron microscopy, can provide valuable insights into the structure-property relationships and guide the design of high-performance materials.

Development of multi-scale computational modeling and simulation tools to predict and optimize the hydrogen storage properties of magnesium-based materials. The integration of first-principles calculations, molecular dynamics simulations, and finite element analysis can provide a comprehensive understanding of the hydrogen storage mechanisms and help to identify the key factors influencing the performance.

Exploration of novel preparation methods and processing techniques to produce magnesium-based materials with unique nanostructures and properties. The combination of different preparation methods, such as ball milling and MWCVD, or the development of new methods, such as 3D printing and self-assembly, can lead to the discovery of innovative materials with superior hydrogen storage performance.

Investigation of the long-term cycling stability and safety of magnesium-based hydrogen storage materials under realistic operating conditions. The development of standardized testing protocols and safety assessment methods, and the establishment of failure mechanisms and mitigation strategies, are crucial for the practical application of these materials.

Optimization of the system design and integration of magnesium-based hydrogen storage materials into practical hydrogen storage and delivery systems. The development of efficient heat management strategies, pressure control systems, and gas purification technologies is essential for the successful implementation of magnesium-based materials in hydrogen fuel cell vehicles and stationary storage applications.

Establishment of large-scale production and recycling processes for magnesium-based hydrogen storage materials. The development of cost-effective and environmentally friendly methods for the synthesis, processing, and recycling of these materials is crucial for their sustainable production and use.

In conclusion, the preparation methods of magnesium-based hydrogen storage materials have undergone significant advancements in recent years, leading to the development of materials with improved hydrogen storage capacity, kinetics, and cycling stability. However, further research efforts are needed to address the existing challenges and realize the practical application of these materials in hydrogen storage and transportation systems. The future research directions and development trends, as outlined in this review, provide a roadmap for the development of high-performance, safe, and cost-effective magnesium-based hydrogen storage materials for a sustainable hydrogen economy.

## Figures and Tables

**Table 1:** Other important properties of magnesium-based hydrogen storage materials and their influencing factors [21,22,23].

Property	Influencing Factors	Effect on Hydrogen Storage Performance
Thermal conductivity	Addition of high-conductivity additives Optimization of system design	Enhances heat transfer during absorption/desorption Improves kinetics and reversibility
Mechanical stability	Incorporation of reinforcing agents Optimization of microstructure	Prevents degradation during cycling Improves long term stability and reliability
Resistance to Impurities	Surface modification and protection Purification of hydrogen gas	Minimizes contamination and degradation Maintains high storage capacity and kinetics
Activation energy	Catalytic additives Nanostructuring	Lowers energy barrier for hydrogen sorption Enhances kinetics and reduces operating temperature

**Table 2:** Key parameters in plasma-assisted ball milling and their effects on the hydrogen storage properties of magnesium-based materials [66,67,68].

Parameter	Effects	Optimized Range
Plasma power	Higher power leads to more intense surface modification Excessive power may cause Sûrface damage	50–200 W
Gas composition	Hydrogen-containing atmosphere promotes hydrogenation Inert gases (e.g., Ar, He) prevent oxidation	H_2_/Ar or H_2_/He ratio: 1:1 to 1:5
Treatment time	Longer time leads to more extensive surface modification Excessive time may cause surface saturation and degradation	10–60 min
Ball-to-powder ratio	Higher ratio leads to more intense milling and surface modification Excessive ratio may cause contamination and amorphization	10:1 to 50:1
Milling speed	Higher speed leads to more intense milling and surface modification Excessive speed may cause overheating and phase transformation	200–600 rpm

**Table 3:** Comparison of different organic ligands used in the organic ligand-assisted synthesis of magnesium-based hydrogen storage materials [82,83,84,85].

Organic Ligand	Role	Effect on Nanostructure	Effect on Hydrogen Storage Properties
Oleylamine	Surfactant Capping agent	Controls particle size and shape Prevents agglomeration	Enhances kinetics and storage capacity Improves cycling stability
Oleic acid	Surfactant Capping agent	Controls particle size and shape Prevents oxidation	Enhances kinetics and storage capacity Improves resistance to air and moisture
Polyvinylpyrrolidone (PVP)	Stabilizer Structure-directing agent	Controls particle size and morphology Improves dispersibility	Enhances kinetics and storage capacity Improves cycling stability
Citric acid	Chelating agent Reducing agent	Controls particle size and shape Provides carbon coating	Enhances kinetics and storage capacity Improves thermal conductivity

**Table 4:** Advantages and disadvantages of different preparation methods for magnesium-based hydrogen storage materials. [100,101,102,103].

Preparation Method	Advantages	Disadvantages
Mechanical ball milling	Simple and scalable process Effective in reducing particle size and creating defects	Long milling times required Potential contamination from milling tools
Methanol-wrapped chemical vapor deposition	Controlled morphology and composition High surface area and improved kinetics	Complex setup and process control Limited scalability
Plasma-assisted ball milling	Enhanced surface activation and modification Improved kinetics and reduced temperatures	Additional cost and complexity of plasma setup Potential plasma instability
Organic ligand-assisted synthesis	Tailored nanostructures and surface properties Improved dispersibility and stability	Removal of organic ligands may be challenging Limited hydrogen storage capacity
Rapid solid-phase reaction	Short reaction times and low energy consumption High-purity products	Limited control over particle size and morphology
Shear-induced reaction	Enhanced solid-state reactions and reduced temperatures Scalable process	Potential contamination from the extrusion or milling tools
Electrochemically assisted synthesis	Control over composition, morphology, and thickness Improved kinetics and reversibility	Complex setup and process control Limited scalability

**Table 5:** Future research directions and strategies for the development of magnesium-based hydrogen storage materials.

Research Direction	Strategy	Expected Outcome
Novel catalytic systems	Explore bimetallic alloys, metal oxides, and MOFs Optimize composition, size, and distribution	Enhanced kinetics and reduced operating temperature Improved hydrogen storage capacity
Advanced characterization techniques	In situ X-ray diffraction, neutron scattering, and TEM Investigate structure–property relationships	Better understanding of hydrogen storage mechanisms Guided design of high-performance materials
Multi-scale computational modeling	Integrate first-principles calculations, molecular dynamics, and finite element analysis Predict and optimize hydrogen storage properties	Accelerated discovery and optimization of materials Reduced experimental trial and error
Innovative preparation methods	Combine different methods (e.g., ball milling + MWCVD) Develop new methods (e.g., 3D printing, self-assembly)	Novel materials with unique nanostructures and properties Enhanced hydrogen storage performance
System design and integration	Develop efficient heat management, pressure control, and gas purification systems Optimize system design for practical applications	Improved overall performance and efficiency Accelerated commercialization of hydrogen storage technologies

## Data Availability

No new data were created or analyzed in this study. Data sharing is not applicable to this article.
